# Reduced grazing pressure delivers production and environmental benefits for the typical steppe of north China

**DOI:** 10.1038/srep16434

**Published:** 2015-11-10

**Authors:** Yingjun Zhang, Ding Huang, Warwick B. Badgery, David R. Kemp, Wenqing Chen, Xiaoya Wang, Nan Liu

**Affiliations:** 1 Department of Grassland Science, China Agricultural University, Beijing 100193, People’s Republic of China; 2 New South Wales Department of Primary Industries, Orange Agricultural Institute, Orange, NSW 2800, Australia; 3 Graham Centre for Agricultural Innovation, Charles Sturt University, Orange, NSW 2800, Australia

## Abstract

Degradation by overgrazing is common in many areas of the world and optimising grassland functions depends upon finding suitable grazing tactics. This four-year study on the northern China steppe investigated combinations of rest, moderate or heavy grazing pressure early in the summer growing season, then moderate or heavy grazing in the mid and late season. Results showed that moderate grazing pressure (~550 sheep equivalent (SE) grazing days ha^−1^ year^−1^) gave the optimal balance between maintaining a productive and diverse grassland, a profitable livestock system, and greenhouse gas mitigation. Further analyses identified that more conservative stocking (~400 SE grazing days ha^−1^ year^−1^) maintained a desirable *Leymus chinensis* composition and achieved a higher live weight gain of sheep. Early summer rest best maintained a desirable grassland composition, but had few other benefits and reduced incomes. These findings demonstrate that reducing grazing pressure to half the current district stocking rates can deliver improved ecosystem services (lower greenhouse gases and improved grassland composition) while sustaining herder incomes.

Grasslands represent 40% of the world land area^1^ and 68% of the world’s grassland are located in developing countries^2^. Many of these grasslands have long been utilised with transhumance grazing systems – a system still used across large parts of Asia and Africa^3^. Population pressures now mean that more settled systems using higher stocking rates, are increasingly common and degradation is widely acknowledged. More than 90% of grasslands in China are considered degraded^4^ by overgrazing because of an increasing number of people dependent on them since the 1950’s^5^. Similar problems are reported throughout Central Asia on the vast Eurasian grasslands^6^.

Productivity of plants and animals, grassland composition and ecosystem services (carbon cycling) are three key variables to be managed when grazing grasslands. Optimising the interaction between them is a key challenge that needs to be addressed^7^. However, maximising individual factors may not be appropriate^8^ and the interactions between key components need to be considered. Over-grazing can reduce the dominance of many desirable species in the grassland^9^ but the greatly reduced animal numbers required to improve grassland composition may be detrimental to net household incomes. Furthermore, recent studies have shown conflicting results about the optimal grazing pressure for greenhouse gas (GHG)^10^ sequestration and few studies have examined this issue at a grazing systems level. These three issues are rarely investigated together because the agronomic discipline focus on productivity and the ecological discipline focus on diversity and species conservation are rarely integrated, yet the application of both are needed to find optimal solutions^11^,^12^.

Since the end of the 20^th^ century, to conserve grasslands, mitigate degradation, and promote economic development in pastoral regions, the Chinese Government has implemented “Control grazing for grassland recovery”, “Grassland ecological compensation incentive mechanism” and a series of other policies and programs to restore grassland ecosystem functions. The key techniques adopted by these policies and programs involve partial or total rests (bans) from grazing that may last for several years, the regulation and control of grazing pressure, and the cessation of transhumance grazing systems. However, it is unclear how these policies designed to provide benefits to the grassland environment also affect households as limited monitoring has been done. The research done, upon which policies are based, used a constant grazing intensity (e.g. light, moderate and heavy grazing) throughout the whole grass growing season to investigate grazing pressure effects on grassland ecosystems^12^ (note: across northern and western China and through central Asia, temperatures limit grassland growth to 3–4 months of summer; fortunately that is when most of the annual rain falls), whereas the forage supply changes dynamically throughout the short growing season (low temperatures limit growth for nine months of the year) while animal food demand is relatively more constant^13^.

Improved grassland management needs to be based on understanding the effects of varying seasonal grazing pressure on productivity and species interactions. Periods of rest, reduced grazing and intense grazing, applied strategically through the year, are the primary tools available to herders to manipulate grasslands to enable recovery of desirable species, to enhance productivity (forage quantity and quality for animal production) and to achieve a state where environmental values are enhanced. Grasslands can sequester soil carbon^14^ and degraded grasslands offer the prospect of storing carbon as part of rehabilitation. The amount sequestered is uncertain because of high variation across different grassland systems^15^. The vast areas of grasslands mean that much carbon could be stored even if the rate of storage per unit area is low. Furthermore, methane (CH_4_) emissions, an important GHG, with 25 times the global warming potential of CO_2_ over 100 year period^16^, can be reduced by improving livestock management^17^. The major challenge for China and throughout the Eurasian grasslands is to identify practices that maintain or restore plant species diversity so that grassland productivity and sustainability is optimised. Assessments need to consider impacts on the grassland and household incomes from changing practices.

This paper details a comprehensive study done to investigate the impact of variable grazing pressure on the productivity of the grassland and animals, grassland composition and GHG mitigation for a native *Leymus chinensis* (typical steppe) grassland in northern China. We tested the hypotheses that reducing grazing pressure from high (district average) to moderate grazing pressure (30% reduction) and early season rest can (i) improve vegetation growth, provide herbage mass that does not restrict intake during grazing, and increase lamb weight gains and gross margins, (ii) maintain *L. chinensis*, the dominant desirable perennial grass, and prevent the encroachment of less desirable forbs and *Artemisia* species in the grassland, and (iii) enhance mitigation of GHGs by increasing soil carbon, CH_4_ uptake by the soil and reducing enteric CH_4_ emissions ha^−1^. A fourth hypothesis tested whether optimum profitability from livestock production is compatible with benefits to the environment of improved grassland composition and enhanced GHG mitigation. The treatments were designed to generate variability in the system so that the underlying mechanisms could be investigated. To understand the underlying relationships that applied across all treatments and to identify the optimal stocking capacity of the grassland, data from across all treatments were combined and regression and multivariate methods used.

## Results

### Grazing pressure influences grassland and animal production

**Figure 1:**
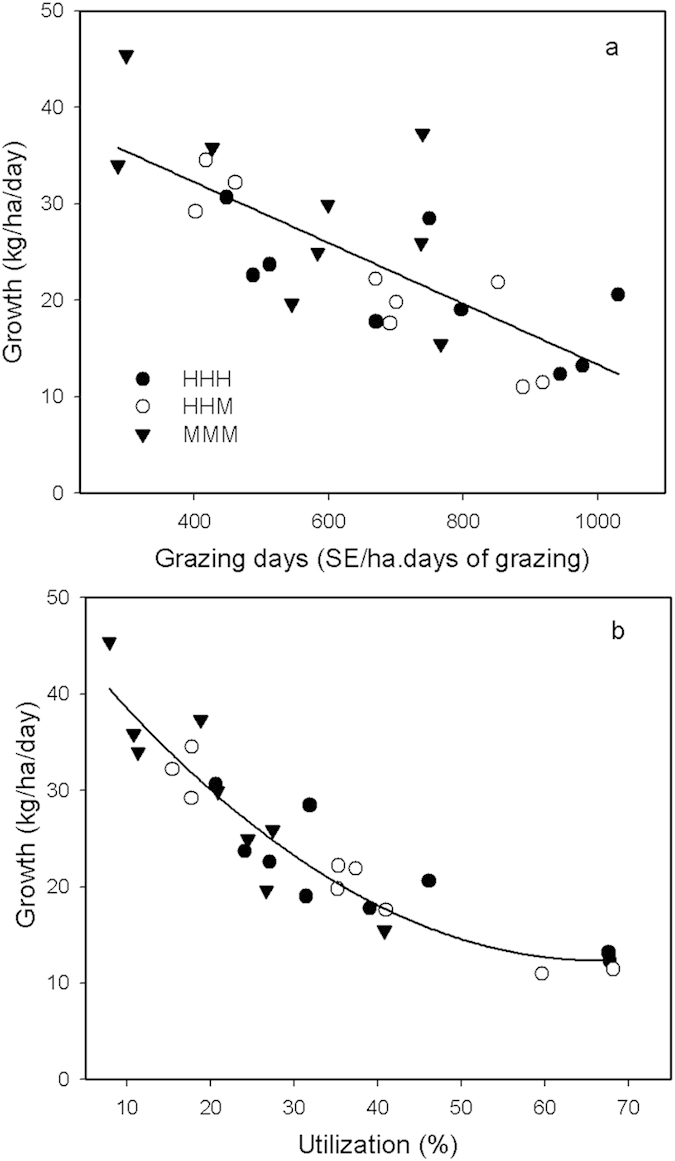
The relationship between average vegetation growth (kg DM ha^−1^ day^−1^) and (**a**) grazing pressure measured as SE grazing days ha^−1^ year^−1^ (1 SE = a 50 kg reference weight animal; Y = −0.03154X + 44.86, Adj R^2^ = 0.55); and (**b**) utilization of above ground herbage mass due to grazing (Y = 8.84 + 44.54*0.0219^X^, Adj R^2^ = 0.87) for season long grazing treatments between 2011 and 2013. Growth rate and utilisation were not calculated for rest treatments.

**Figure 2:**
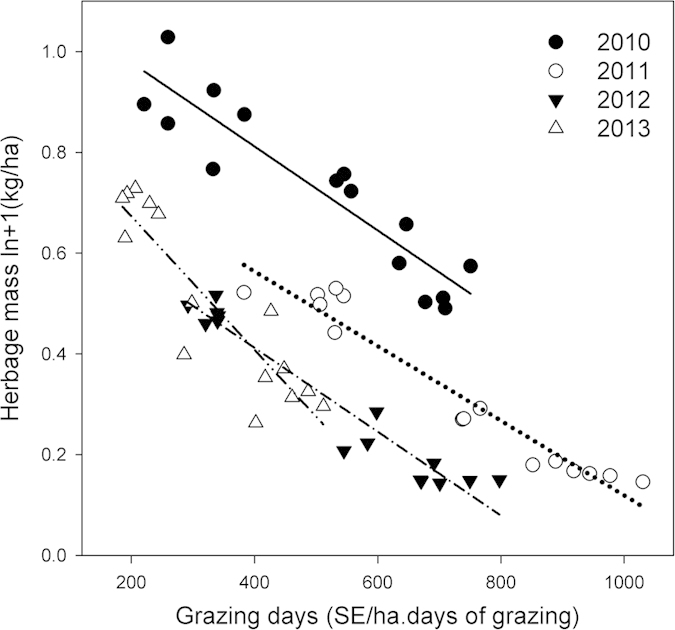
The relationship between average grazing season herbage mass ln +1 (kg DM ha^−1^) and SE grazing days ha^−1^ year^−1^ (1 SE = a 50 kg reference weight animal); Adj R^2^ = 0.94) between 2010 and 2013 (2010, Y = −0.00083X + 1.1451; 2011, Y = −0.00074X + 0.86; 2012, Y = −0.00083X + 0.7452; 2013, Y = −0.0013X + 0.9387).

Stocking rates varied through the early, mid and late periods of the summer grazing season and ranged from rests (R) to moderate (M) to high (H: district average for the region) (Supplementary Tables 1 and 2). The average net growth of above-ground vegetation in the season-long grazing treatments from 2011 to 2013 was 50% higher in MMM (30 kg DM ha^−1^ day^−1^, averaged throughout the grazing season) than the HHH/HHM (21–22 kg DM ha^−1^ day^−1^) treatments (*P* = 0.003). An integrated measure of the grazing pressure in each treatment was calculated as the sheep equivalent (SE) grazing days ha^−1^ year^−1^, which took into account the actual SE ha^−1^, variation in the duration of each grazing period and variation in these terms between years (Supplementary Table 1). There was a strong association between SE grazing days ha^−1^ year^−1^, the growth rate of the grassland (Fig. 1a), and the proportion of the vegetation utilised by the sheep (Fig. 1b). The average pasture herbage mass (standing biomass) (Fig. 2), was related to SE grazing days ha^−1^ year^−1^, but there were different relationships between years, with the herbage mass highest in 2010. The slope of the relationship increased in 2013 with the herbage mass increasing more under lower SE grazing days ha^−1^ year^−1^ indicating improved recovery of productivity.

**Figure 3:**
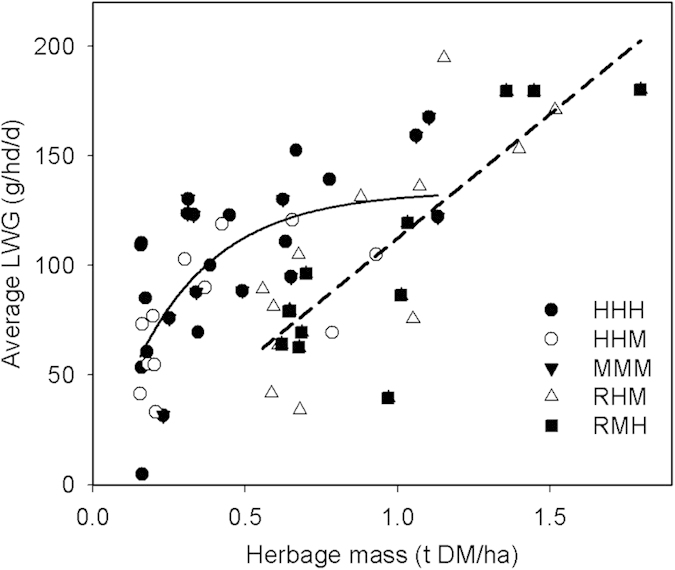
The average liveweight gain (LWG) per head (g hd^−1^ day^−1^) compared to herbage mass for season-long grazing (__; Y = 136.8 – 126.8(0.0391X); Adj R2 = 0.44) and rest (_ _; Y = 113.1X – 0.7; Adj R2 = 0.64) grazing treatments.

Animal productivity, measured as liveweight gain (LWG), showed a similar response over the grazing season in each of the four years (2010–2013). The LWG per head for all sheep was greatest in the early (121.4 ± 12.9 g hd^−1^ day^−1^) and mid (113.5 ± 7.0 g hd^−1^ day^−1^) periods of summer, then declined significantly in the late summer (47.5 ± 11.1 g hd^−1^ day^−1^) (*P* < 0.001) as plants completed their reproductive phase and frosts commenced. There were two relationships between average LWG per head and herbage mass across all treatments (Fig. 3). The season-long grazing treatments (HHH, HHM and MMM) showed the typical response curve expected where intake and production are limited by the quantity of available pasture^18^ with LWG decreasing substantially when the herbage mass declined below 0.5 t DM ha^−1^. In the early summer rest treatments, herbage mass and LWG per head was highest in 2010 (points at the top right of Fig. 3) but when herbage mass was <1 t DM ha^−1^ LWG per head was lower than other treatments probably due to lower forage quality (not measured) which is a consequence of leaves being older and more stem growth when grazing started in summer. The average LWG ha^−1^ year^−1^ over the grazing season was highest for the HHH (79.1 kg ha^−1^ year^−1^), HHM (59.6 kg ha^−1^ year^−1^) and MMM (67.8 kg ha^−1^ year^−1^) treatments and least for the rest treatments (RHM = 34.1 kg ha^−1^ year^−1^, RMH = 31.0 kg ha^−1^ year^−1^) (*P* < 0.001).

Livestock profitability is important for herders as they are among the poorest people in China. Gross margins were derived from the total LWG ha^−1^ year^−1^ to assess the value of summer grazing. The HHH (853 ± 119 ¥ ha^−1^), HHM (639 ± 59 ¥ ha^−1^) and MMM (733 ± 95 ¥ ha^−1^) treatments resulted in significantly higher gross margins than the RHM (365 ± 47 ¥ ha^−1^) and RMH (332 ± 119 ¥ ha^−1^) treatments (*P* < 0.001). Ultimately the cost of pen feeding in winter will determine the profitability of these systems^19^, which would be more costly for early season rest treatments. On average there was a 30% higher stocking rate (SE ha^−1^) in HHH compared to MMM, though the gross margins for summer grazing were not significantly different, but as winter feeding costs in HHH would be greater due to more animals the overall profitability of higher stocking rate systems is likely be lower than for moderate stocking rates. The maximum LWG for continuously grazed treatments was 127 g hd^−1^ day^−1^. Financial analyses^10^ often show that the optimal gross margin is about 75% of that biological maximum *i.e.* 95 g hd^−1^ day^−1^, which was associated with an average summer herbage mass of above 0.5 t DM ha^−1^.

### Grassland composition change under different grazing management

**Figure 4:**
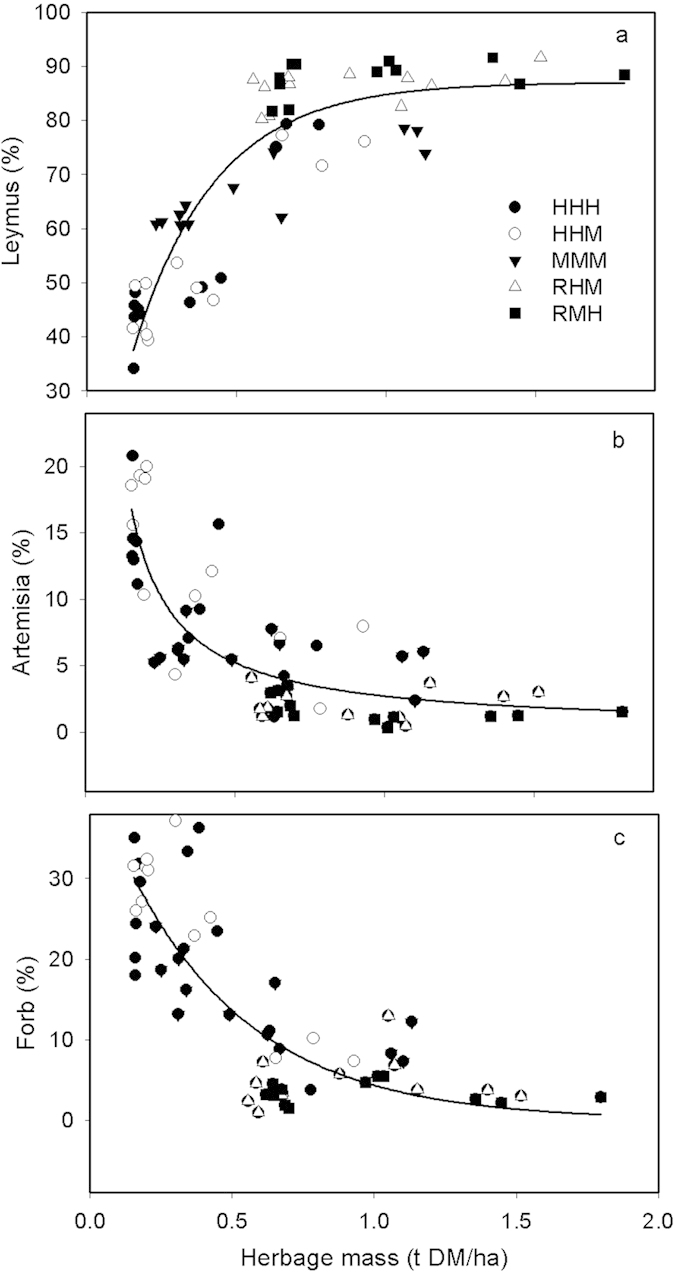
The average proportion of (**a**) *Leymus chinensis* (Y = 0.9035−0.7475(0.067^X^); Adj R^2^ = 0.80), (**b**) *Artemisia scopari* (Y = 0.02464 + 0.2865(0.00803^X^); Adj R^2^ = 0.68) and c) forbs (Y = 0.0212 + 0.4307(0.0666X); Adj R2 = 0.68) throughout the year compared to the average herbage mass.

The plant species found in the experimental fields are shown in Supplementary Table 3. The initial proportion and biomass of the desirable perennial grass *L. chinensis* was high in 2010; 89% in early season rests to ~77% in other treatments, then declined over time in response to the continuously grazed treatments. After 2011 the dominance of *L. chinensis* was reduced in HHH/HHM to ~46% and to ~64% in MMM but remained ~87% in the early season rest treatments (*P* < 0.001). The proportion of the less-desirable species *Artemisia* sp. and forbs showed the opposite results being least in the early season treatments, moderate in MMM and highest in the high stocking rate treatments (*P* < 0.001). These relationships are best illustrated in relation to changes in herbage mass (Fig. 4). There was an exponential decrease in the proportion of *L. chinensis*, declining below ~1 t DM ha^−1^ of herbage mass (Fig. 4a) and below 70% when the herbage mass was < 0.5 t DM ha^−1^. In contrast (Fig. 4b,c), *Artemisia* sp. increased above 5% when the herbage mass was below ~0.5 t DM ha^−1^ and forbs increasing above 10% at the same point. Some medium-term resilience was evident in the grasslands, when stocking rates were reduced in 2013 (Supplementary Figs 1 and 2) both *L. chinensis* and *Artemisia* sp. significantly reversed treatment trends (*P* < 0.001) with reduced grazing pressure. Forbs did not decrease significantly in the final year. Rest treatments maintained similar, desirable, proportions of plant species across all four years.

### Grazing pressure controls carbon sequestration

Grassland management affected the GHG balance from livestock and the soil. The heavily grazed treatments (HHH, HHM) had significantly higher CH_4_ emissions from animals over the grazing season (23.3 and 21.4 kg ha^−1^ year^−1^), compared to MMM (16.8 kg ha^−1^ year^−1^) and early season rest treatments (RMH and RHM, 13.6 and 15.2 kg ha^−1^ year^−1^, respectively) (*P* < 0.001). CH_4_ emissions per head increased linearly with LWG of animals (Adj R^2^ = 0.69). Estimates of emission intensity (kg CH_4_ kg LWG^−1^) were higher in the early season rest treatments (RHM, RMH; both 0.50 g CH_4_ kg LWG^−1^) because those treatments were grazed for a shorter period and not when the highest animal growth rates occurred early in the growing season, hence total LWG was less. There were no significant differences in emission intensity between MMM, HHH and HHM (average 0.32 g CH_4_ kg^−1^ LWG) (*P* = 0.001).

Improved grassland management can increase microbial activity, CH_4_ oxidation and soil-atmospheric exchange^17^. CH_4_ uptake into the soil during the grazing season did vary significantly between treatments and was highest in the moderately grazed (MMM) treatment (3.72 mg m^−2^ day^−1^); compared to the other treatments (average 1.46 mg m^−2^ day^−1^)^20^.

There was no difference between treatments in soil organic carbon (SOC) stocks at the start of the experiment (mean 24.6 Mg ha^−1^). However after four years of treatments, sequestration of SOC in MMM (1.18 Mg ha^−1^ year^−1^) was higher than for RHM and RMH (0.61 and 0.57 Mg ha^−1^ year^−1^, respectively) with lowest rates in HHH (0.05 Mg ha^−1^ year^−1^) and HHM (0.12 Mg ha^−1^ year^−1^; not significantly different from RMH) (*P* < 0.001).

The main sources and sinks of GHGs were combined throughout the grazing season to establish the systems level emissions. The MMM treatment had the highest net sequestration of GHGs at 4 t CO_2_-e ha^−1^ year^−1^, while the HHH and HHM actually emitted GHGs (Table 1; *P* < 0.001). This GHG budget does not account for N_2_O, which should be small due to low availability of soil N in this native grassland^21^. The budget could not estimate emissions that occurred outside of the grazing season when no data was collected, a period when CH_4_ fluxes from the soil would be expected to be much less (approximately 35% of the annual emissions^22^) and CH_4_ emissions from sheep would depend on the production system used through winter. Nevertheless, the results demonstrate the potential for conservative grazing management in the MMM, RHM and RMH treatments to sequester reasonable levels of GHGs, mainly through increases to SOC.

### Optimal grazing management is compatible for production and environmental outcomes

**Figure 5:**
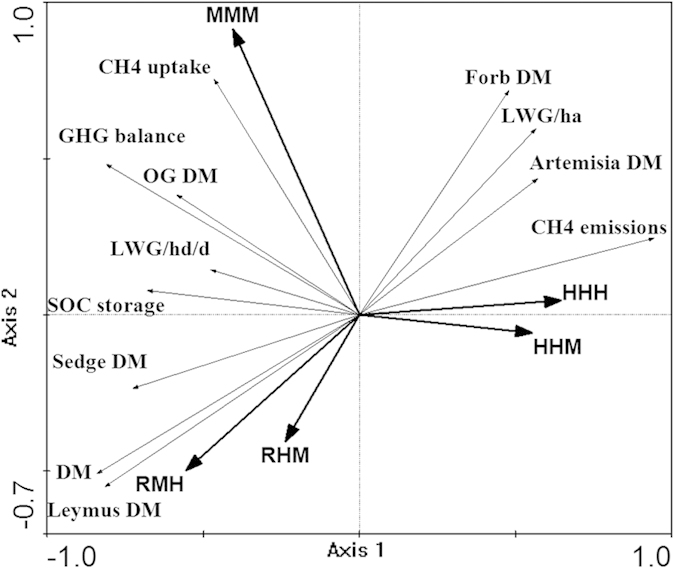
Partial redundancy analysis (RDA) of field data in axis1 × aixs_2_ ordination planes constrained by the five treatments.

Redundancy Analysis (RDA) that included the main factors measured showed that the first axis was related to overall grazing pressure while the second axis separated treatments differing in grazing pressure early in the season (axis 1 and axis 2 explained 52.3% and 16.3% of the variance respectively, total 68.6%; F = 10.76; *P* = 0.004; Fig. 5). The desirable outcomes lay between the RMH/RHM and MMM treatments. The MMM treatment had significant positive effects on CH_4_ uptake (kg CO_2_-e ha^−1^ year^−1^), forb DM, LWG per head, and overall GHG balance (kg CO_2_-e ha^−1^ year^−1^). The two early season rest treatments had larger positive effects on *L. chinensis*, the dominant species, and on the herbage mass of the grassland. The two heavy grazing treatments had a large effect on CH_4_ emissions from sheep (kg CO_2_-e ha^−1^ year^−1^) and a negative effect SOC storage (kg CO_2_-e ha^−1^ year^−1^) and were associated with increasing less-desirable species. Overall, treatments that positively influenced species composition were also associated with benefits to CH_4_ uptake and SOC storage.

## Discussion

This research is the first to demonstrate that profitable grazing systems can deliver enhanced environmental outcomes, through improved grassland composition and sequestration of greenhouse gases, for a site representative of the typical steppe of northern China. These findings suggest that current stocking rates could be reduced considerably and the use of bans early in the summer growing season may not be desirable in this environment when all aspects of the grazing system are considered.

To address degradation the Chinese Government has implemented a series of policies and programs to restore grassland ecosystem functions^23^. These policies generally limit early season grazing and can impose total grazing bans for up to five years, to try and restore grasslands to a desirable state. While winter grazing is practiced in many regions across China, it was excluded from this experiment because it is counterproductive for livestock production compared to pen feeding^24^ and may do additional damage to grasslands. The results presented here indicate that sustainable solutions for managing grasslands are feasible and viable for herders at this site.

Stocking rates have been the criteria used to define grazing pressures, but they fail to consider variable periods for grazing as can apply with the use of tactical rests, or rotation of livestock around the landscape. The analyses presented here found that better relationships were obtained by considering grazing pressure as SE grazing days ha^−1^ year^−1^ (stocking rate data in Supplementary Information Table 1). That criterion allows some flexibility as herders could then adjust animal numbers through a season to achieve the targeted SE grazing days ha^−1^ year^−1^. It may prove to be a better criterion for setting stock numbers by local authorities, though further research is needed to test this approach.

At this site moderate stocking throughout the summer grazing season (~550 SE grazing days ha^−1^ year^−1^; 5.6 SE ha^−1^) was the treatment that came closest to producing the best balance between maintaining a productive grassland, desirable plant species, a profitable livestock system, and mitigating greenhouse gases through increased soil carbon^25^, CH_4_ uptake by the soil^17^ and efficient CH_4_ emissions per unit of weight gain^17^. The early season rest treatments best maintained a desirable grassland composition, supporting the government’s policies on early season rest, but when a more holistic view is taken they did not provide as many benefits in other areas and they place an onus on the government to provide economic support for households to facilitate these rests. Considerable extra stored fodder would be required to sustain early season rests and that is likely to be uneconomic for herders. Moderate continuous grazing during the grazing season from June to October is a better way to manage grasslands sustainably in this environment as this could maintain, or enhance the incomes of herders. Note however that very early season grazing (before June) was excluded from this experiment and is likely be detrimental to many of the factors measured.

The data presented can be used to define an optimal moderate grazing strategy for the typical steppe and the minimum herbage mass for management. As herder livelihoods are a core issue, the financially optimal LWG was about 100 g hd^−1^ day^−1^, which occurred when the herbage mass was maintained above 0.5 t DM ha^−1^ through the grazing period (Fig. 3). This herbage mass was associated with approximately 400 SE grazing days ha^−1^ year^−1^ (Fig. 2); less than the MMM treatment applied. In turn those values equated to near peak grassland growth rates (Fig. 1a) a utilization rate of about 20% (Fig. 1b) a *L. chinensis* proportion of 70% (Fig. 4a) *Artemisia* of 5% (Fig. 4b) and forbs of 12% (Fig. 4c), all of which are reasonable criteria for a sustainable grassland. It would be reasonable to assume that the trends in CH_4_ uptake and SOC storage for MMM would also apply to a lower continuous summer grazing pressure of 400 SE grazing days ha^−1^ year^−1^.

The early season rest treatments showed variable results based on the length of grazing periods. Some refinement of early season rest strategies may be possible, though this study does not give definitive solutions. Rather than setting a time limit for rests, it could be preferable to set a value of herbage mass that needs to be reached before grazing commences. Maintaining herbage mass above 0.5 t DM ha^−1^ optimised pastoral outcomes and suggests that rests aiming to achieve that target before grazing starts may be a preferable tactic to a time-based rest. Setting such a target for herbage mass would help reduce dust storms in early summer, which are common across northern China and adjusts for variable timing of when rainfall commences.

Higher levels of herbage mass with moderate grazing pressure enable animals to select better quality diets, especially when management has achieved a dominance of desirable plant species. The criteria considered here may not be suitable to return severely degraded grasslands to a desirable composition with reduced grazing pressure^26^,^27^. In such cases other rehabilitation strategies, such as reseeding and long-term grazing exclusion^28^, may need to be considered. Fortunately, severe degradation only applies to 10% of China’s grasslands^23^.

An average moderate grazing pressure of 400 SE grazing days ha^−1^ year^−1^ represents about half the current district stocking rates^29^ over the growing season in the study area. Financial analyses showed similar reductions in stocking rates could result in higher net financial returns to households^5^,^11^ for a range of other grassland environments. Those other studies did not evaluate ecological or environmental benefits. This moderate grazing pressure would result in animal’s utilising (consuming) about 20% of the grass grown in the growing season. Lower utilisation rates could be anticipated in less productive environments and higher in more productive. Actual losses of plant material over the growing season could be 2-3 times this value as these calculations do not include normal plant and tissue death rates, losses from micro and meso-organisms and physical damage from grazing livestock.

The gross margins in this study did not take into account government payments or winter feeding, but it is unlikely those factors would modify these results. Over the longer-term market forces for products and environmental services may generate self-sustaining changes to management. Previous studies^5^,^30^ have demonstrated the role of market forces in determining the financially optimal stocking rates in desert steppe. Market based instruments (MBIs) are increasingly being used as a mechanism to pay farmers for environmental services and these markets are developing for GHG in agriculture and in the future may provide incentives to improved management of grasslands in China. This study highlights that management practices to improve the profitability of livestock systems and to deliver enhanced ecosystem services can align. While there are many challenges in designing an MBI for soil carbon^31^, there are other offsets in enhanced CH_4_ uptake by the soil and potentially lower emissions intensity per unit of product to be considered in an incentive scheme and these systems could be based around maintaining a minimum herbage mass above 0.5 t DM ha^−1^.

Previous studies have found that increased grazing pressure reduce grassland diversity^32^, soil carbon^33^ and CH_4_ mitigation^17^. However, for this site in the typical steppe moderate grazing pressure provided the greatest benefits to these ecosystem services and financially optimised livestock production, indicating that production and the environmental benefits can be achieved simultaneously. While moderate stocking rates have been demonstrated to achieve production benefits^11^, the scale of environmental benefits from this type of management has not been previously documented simultaneously.

## Methods

All the animals used in the study received the approval of the China Agricultural University

Laboratory Animal Care Advisory Committee, and all methods were carried out in accordance with the approved guidelines.

### Site

The experiment was conducted at Guyuan, in Hebei province, China (41°45′N 115°39′E). The area has a semi-arid continental monsoon climate with an average annual precipitation of 430 mm, mostly occurring between July and September. The altitude is 1430 m above sea level and the annual mean temperature is 1.4 °C. *L. chinensis*, *Artemisia* sp. and *Carex duriuscula* were the dominant species in the grassland^34^. The grazing seasons are approximately: early summer– middle of June to the middle of July, mid summer– middle of July to the middle of September, autumn from the middle of September to early October (Supplementary Table 2).

### Experiment design

The experimental design has been previously reported^20^,^35^. The core design of this grazing experiment involved combinations of rest (R), moderate (M) and heavy (H) grazing pressure in the early summer season, then moderate or heavy grazing in the mid and late season. To test the 27 interactions between all components of those livestock management systems was unmanageable, not all were relevant and hence some compromises were made. Five grazing treatments were implemented: RMH, RHM, MMM, HHM and HHH through early, mid and late summer. Chinese government policy to reduce grazing pressure and address environmental problems induced by overgrazing meant no grazing was possible before June, hence treatments started after that time. There were 3 replicates for each treatment in a completely random design, requiring a total of 15 plots each 1.5 ha in size. The experiment was for four years and a core group of animal’s remained within each treatment throughout the year, new animals were used each year. Animal numbers were added or removed throughout the year as required to apply R, M, and H treatments. Stocking rates for H were the district average and M was 30% less, an estimate initially considered to be close to the optimal grazing pressure. Early season rests are promoted to enhance grassland composition and these treatments (RHM and RMH) were chosen for this experiment. A final treatment was added to determine whether decreasing grazing pressure late in the season was beneficial (HHM).

### Animals and management

At the beginning of each year Mongolian fat-tail sheep (<2 years old) of similar live weight (average starting weight 36–39 kg) were randomly allocated to 15 experimental plots. There were ten sheep per M plot (6.7 ha^−1^) and 14 per H plot (H 9.3 ha^−1^) from 2010 to 2012, which was reduced to 7 (4.7 ha^−1^) and 11 (H 7.3 ha^−1^) sheep in 2013, as it was apparent that grazing pressure was high in the two previous years resulting in very high utilisation of aboveground vegetation. For analyses, stocking rates were standardized to a 50 kg sheep equivalent (SE) using actual liveweights. The length of grazing periods for each season varied from 29–30 day in the early period, 27–60 day in mid period and 20–32 day in the late period, depending upon year. The overall grazing pressure was calculated as grazing days (SE grazing days ha^−1^ year^−1^) (Supplementary Table 1). Sheep grazed plots continuously, and there was a sheepfold for them to shelter. Water and salt was available at all times and no feed supplement was given.

### Grassland condition measurements

Herbage biomass was determined at the end of the early, mid and late grazing periods. The aboveground vegetation within a 0.5 m × 0.5 m quadrat was cut to ground level at 9 random sites across each plot. After harvesting, the material from each quadrat was divided into different species groups: the dominant grass *L. chinensis*, the steppe degradation indicator plant *Artemisia* sp., other grasses, sedges and forbs, then oven dried to a constant weight to determine dry matter (DM) content. Five cages were randomly placed in each plot to exclude sheep grazing and 0.5 m × 0.5 m quadrats cut at the end of each grazing period and the cages were moved to a new location to estimate growth rates^36^.

### Animal growth measurement

The LWG of sheep was measured by weighing each sheep at the start and end of each grazing period. Animal health was monitored at the same time and no health issues were identified.

### Measurements of CH_4_ emission and intake of grazing livestock

CH_4_ emission (g hd^−1^ day^−1^) and pasture intake (kg hd^−1^ day^−1^) of sheep were estimated using the Grazfeed decision support tool^37^. For each plot and grazing period, the average weight, age, estimated mature weight (45 kg), fleece length (2 cm), latitude, the quantity of vegetation and the estimated quality of vegetation (based on stage of growth) were entered into the model. The quality parameter was varied until the predicted LWG reflected the measured LWG^38^. Daily CH_4_ emissions were summed to give the total emission for each grazing period. The LWG for the entire season was divided by the total CH_4_ emissions to determine the emissions intensity (g CH_4_ kg LWG^−1^). Utilisation was estimated as total intake (kg DM ha^−1^ yr^−1^) relative to estimated aboveground vegetation growth (kg DM ha^−1^ yr^−1^) over the entire grazing period in the long grazing treatments (MMM, HHM, HHH, Grassland growth and utilisation were not calculated for early season rest treatments or for data collected in 2010 due to incomplete sampling).

### CH_4_ flux measurements of grassland

CH_4_ flux in grassland soil was measured between 09:00 am and 11:30 am, 30 days before and 20 days after the early season grazing commenced, 20 before and 45 days after mid season grazing and 20 days after later season grazing with a fast Greenhouse Gas Analyzer (LGR, U.S.A.; which used an off-Axis Integrated Cavity Output Spectroscopy (OA-ICOS), for the rate of change in CH_4_ concentration within an opaque chamber; CH_4_ detection precisions were within 1 ppbv at 5 seconds). The flux was calculated^39^ and the methods are fully described elsewhere^20^.

### Soil organic carbon measurement

Soil samples were taken at the beginning and end of the experiment in mid-May 2009 and mid-August 2013. Samples were taken (4 cm diameter soil corer) for the 0-10 cm soil depth increments at nine locations (at each location, three cores were combined for each depth to form one bulk sample) within each of the 15 plots^34^. Samples from each plot were then sieved to 2 mm to remove roots, part of the soil samples were dried at 40 °C for 4 days, ground and analysed for SOC using an autoanalyzer (TOC, Elementar, Germany). The inorganic C was removed from soil sampling with 1 m HCl prior to SOC determination, so the total C concentration was equal to the organic carbon concentration.

### Data analyses

ANOVA and regression analyses was done on the interactions between treatments, year and to test relationships between variables using GENSTAT V16^40^. Because there were differences in grazing pressure between years, the SE grazing days ha^−1^ year^−1^ were standardized (SE grazing days ha^−1^ year^−1^/average SE grazing days ha^−1^ year^−1^) for each treatment and used as a covariate in ANOVA. Data were log_e_-transformed where residuals were not normally distributed. For herbage mass a regression in groups was used to determine changes from 2010. An RDA ordination was used to determine the relationship between multiple variables, using CANOCO 4.5^41^.

## Additional Information

**How to cite this article**: Zhang, Y. *et al.* Reduced grazing pressure delivers production and environmental benefits for the typical steppe of north China. *Sci. Rep.* **5**, 16434; doi: 10.1038/srep16434 (2015).

## Supplementary Material

Supplementary Information

## Figures and Tables

**Table 1:** The average flux of greenhouse gases (GHGs) monitored over the grazing season for each treatment.

GHG fluxes^	lsd	HHH	HHM	MMM	RHM	RMH
SOC sequestration (kg CO_2_-e ha^−1^ yr^−1^)	1156.7^***^	−164.7	−410.1	−4343.0	−2219.9	−2072.2
CH_4_ uptake (kg CO_2_-e ha^−1^ year^−1^)^#^	28.0^**^	−36.6	−34.5	−103.0	−51.2	−39.4
CH_4_ sheep emissions (kg CO_2_-e ha^−1^ year^−1^)	43.9^***^	651.6	597.8	470.7	424.2	380.7
GHG flux (kg CO_2_-e ha^−1^ year^−1^)	1164.9^***^	384.4	92.9	−4014.7	−1886.8	−1767.4

The greenhouse gases fluxes have been standardised as carbon dioxide equivalents (CO_2_-e). Least significant differences (lsd) are presented (**P < 0.01,***P < 0.001).

^^^Where CH_4_ = 25 CO_2_-e; negative values represent sequestration and positive values represent emissions^16^. ^#^Only monitored in 2011 and 2012.
