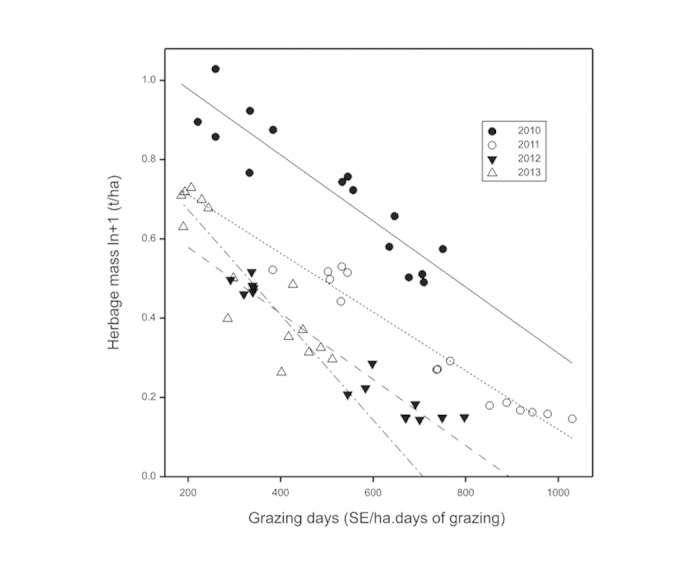# Corrigendum: Reduced grazing pressure delivers production and environmental benefits for the typical steppe of north China

**DOI:** 10.1038/srep19388

**Published:** 2016-01-22

**Authors:** Yingjun Zhang, Ding Huang, Warwick B. Badgery, David R. Kemp, Wenqing Chen, Xiaoya Wang, Nan Liu

Scientific Reports 5: Article number: 1643410.1038/srep16434; published online: 11102015; updated: 01222016

This Article contains an error in Fig. 2, where the y-axis ‘Herbage mass ln + 1(t/ha)’ was incorrectly given as ‘Herbage mass ln + 1(kg/ha)’. The correct Fig. 2 appears below as Fig. 1.

## Figures and Tables

**Figure 1:**