# Supplementary material for: Reduced grazing pressure delivers production and environmental benefits for the typical steppe of north China
Source: Sci Rep. 2015 Nov 10;5:16434. doi: 10.1038/srep16434 (PMC4639777; doi:10.1038/srep16434)

Moderate grazing pressure delivers production and environmental benefits for the steppes of north China

Yingjun ZHANG [a1*], Ding Huang [a1], Warwick B. Badgery [b1], David R. Kemp[c1], Wenqing Chen[a], Xiaoya Wang [a], Nan LIU [a]

[a]Department of Grassland Science, China Agricultural University, Beijing 100193, People's Republic of China

[b]New South Wales Department of Primary Industries, Orange Agricultural Institute, Orange, NSW 2800, Australia;

[c]Graham Centre for Agricultural Innovation, Charles Sturt University, Orange, NSW 2800, Australia

*To whom correspondence should be addressed. E-mail: zhangyj@cau.edu.cn

[1]These authors contributed equally to this work

Supplementary Table 1 Experimental periods and standardised stocking rate (SE ha^{-1}; 1 sheep equivalent (SE) = a 50kg reference weight animal to adjust for different size animals over time) in different seasons each year. The grazing days (SE grazing days ha^{-1} $year^{-1}$) represents the stocking rate x number of days of grazing.

Year	Treatment	Early summer		Mid summer		Late summer		Average	Total	Grazing
		SR	Days	SR	Days	SR	Days	SR	Days	Days
2010	HHH	7.4	36	8.3	34	8.7	32	8.0	90	722
	HHM	7.2	36	7.9	34	6.0	32	7.2	90	651
	MMM	5.5	36	6.3	34	6.6	32	6.1	90	545
	RHM	0.0	0.0	6.8	34	6.0	32	6.5	54	350
	RMH	0.0	0.0	3.6	34	6.2	32	4.6	54	247
2011	HHH	7.3	33	8.2	60	8.7	28	8.1	121	977
	HHM	7.2	33	8.0	60	6.1	28	7.3	121	886
	MMM	5.4	33	6.3	60	6.9	28	6.2	121	748
	RHM	0.0	0.0	6.4	60	5.1	28	6.0	88	526
	RMH	0.0	0.0	4.6	60	7.1	28	5.4	88	474
2012	HHH	6.9	29	7.4	52	7.7	20	7.3	101	739
	HHM	6.9	29	7.4	52	5.2	20	6.8	101	687
	MMM	5.2	29	5.8	52	6.0	20	5.7	101	573
	RHM	0.0	0.0	4.9	52	3.9	20	4.6	72	334
	RMH	0.0	0.0	3.8	52	6.1	20	4.5	72	323
2013	HHH	5.9	29	6.3	27	6.7	21	6.3	77	482
	HHM	5.8	29	6.2	27	4.4	21	5.5	77	427
	MMM	4.1	29	4.3	27	4.7	21	4.3	77	332
	RHM	0.0	0.0	4.8	27	3.2	21	4.1	48	197
	RMH	0.0	0.0	3.5	27	5.9	21	4.6	48	220

Supplementary Table 2 The dates grazing started between years for each summer

Year	Early summer start	Mid summer start	Late summer start	Late summer finish
2010	14/06/2010	21/07/2010	24/08/2010	14/09/2010
2011	9/06/2011	13/07/2011	11/09/2011	10/10/2011
2012	15/06/2012	15/07/2012	5/09/2012	26/09/2012
2013	21/06/2013	21/07/2013	17/08/2013	8/09/2013

Supplementary Table 3 Plant species list and species functional groups found during the experiment.

	Species list
Dominant grass	*Leymus chinensis* (Trin.)Tzvel.
Artemisia	*Artemisia scoparia* Waldst. et Kit.<br>*Artemisia tanacetifolia* Linn.<br>*Artemisia annua* L.<br>*Artemisia frigida* Willd. Sp. Pl.
Sedge	*Carex duriuscula* C.A.Mey.
Other grass	*Phragmites communis* Trin.<br>*Stipa krylovii* Roshev<br>*Cleistogenes squarrosa* (Trin.) Keng<br>*Puccinellia distans*<br>*Leymus secalinus* (Georgi) Tzvel.
Forb	*Inula japonica* Thunb.<br>*Potentilla bifurca* Linn.<br>-Potentilla anserina Linn.<br>*Taraxacum mongolicum* Hand.-Mazz.<br>*Polygonum sibiricum* Laxm.<br>*Plantago major* L.<br>*Iris lactea* Pall. var<br>Astragalus tataricus Franch.<br>*Medicago ruthenica* (Linn.)Trautv.<br>*Heteropappus altaicus* (Willd) Novopokr<br>*Suaeda glauca* (Bunge) Bunge in Bull.<br>*Atriplex patens* (Litw.)Iljin<br>*Lactuca elata* Hemsl.<br>*Saussurea amara* (Linn.) DC

Supplementary Figure 1 The relationship between the proportion of *Leymus chinensis* and herbage mass (kg DM/ha) for each treatment and year.

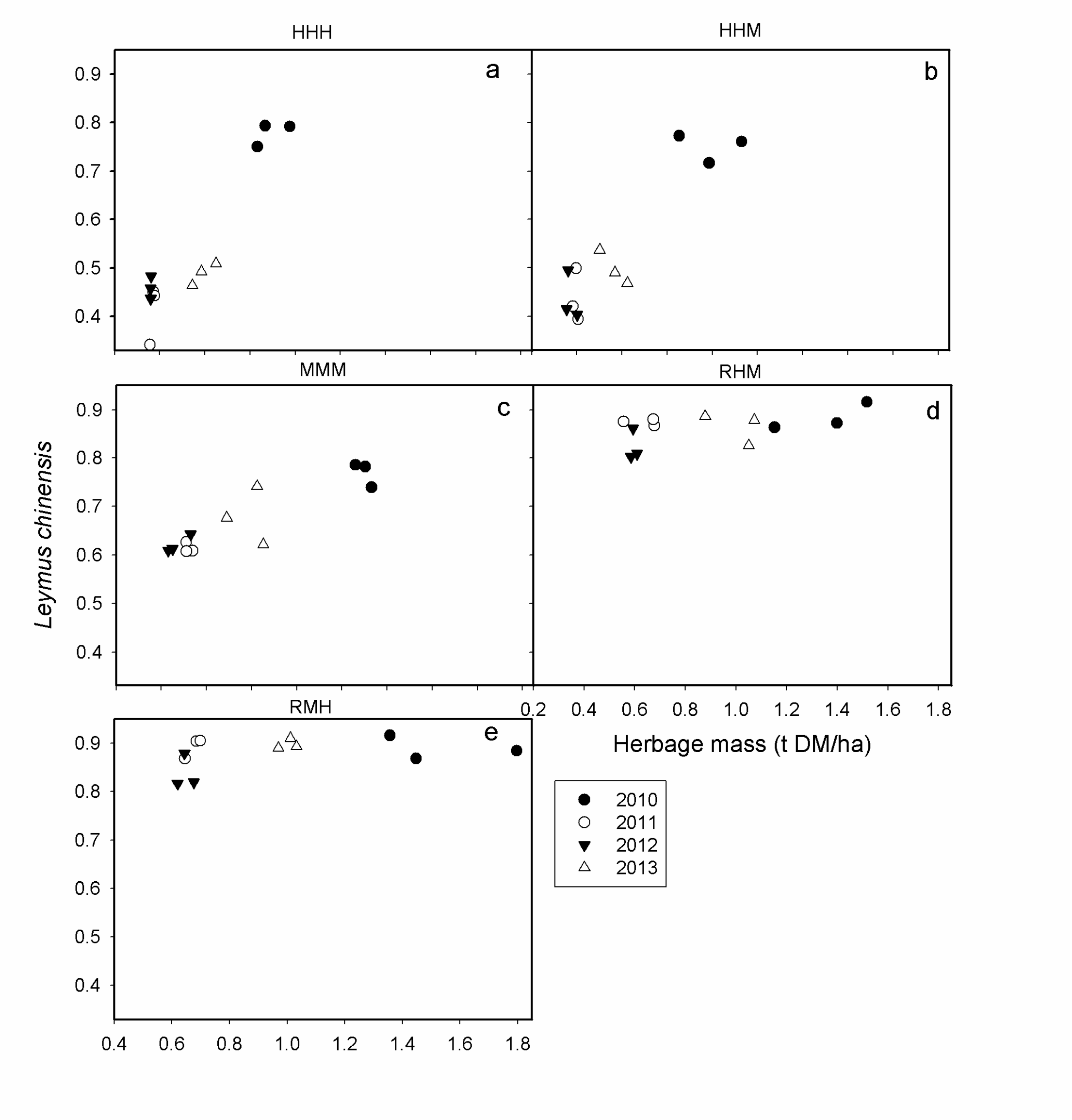

Supplementary Figure 2 The relationship between the proportion of *Artemisia scoparia* and herbage mass (kg DM/ha) for each treatment and year.

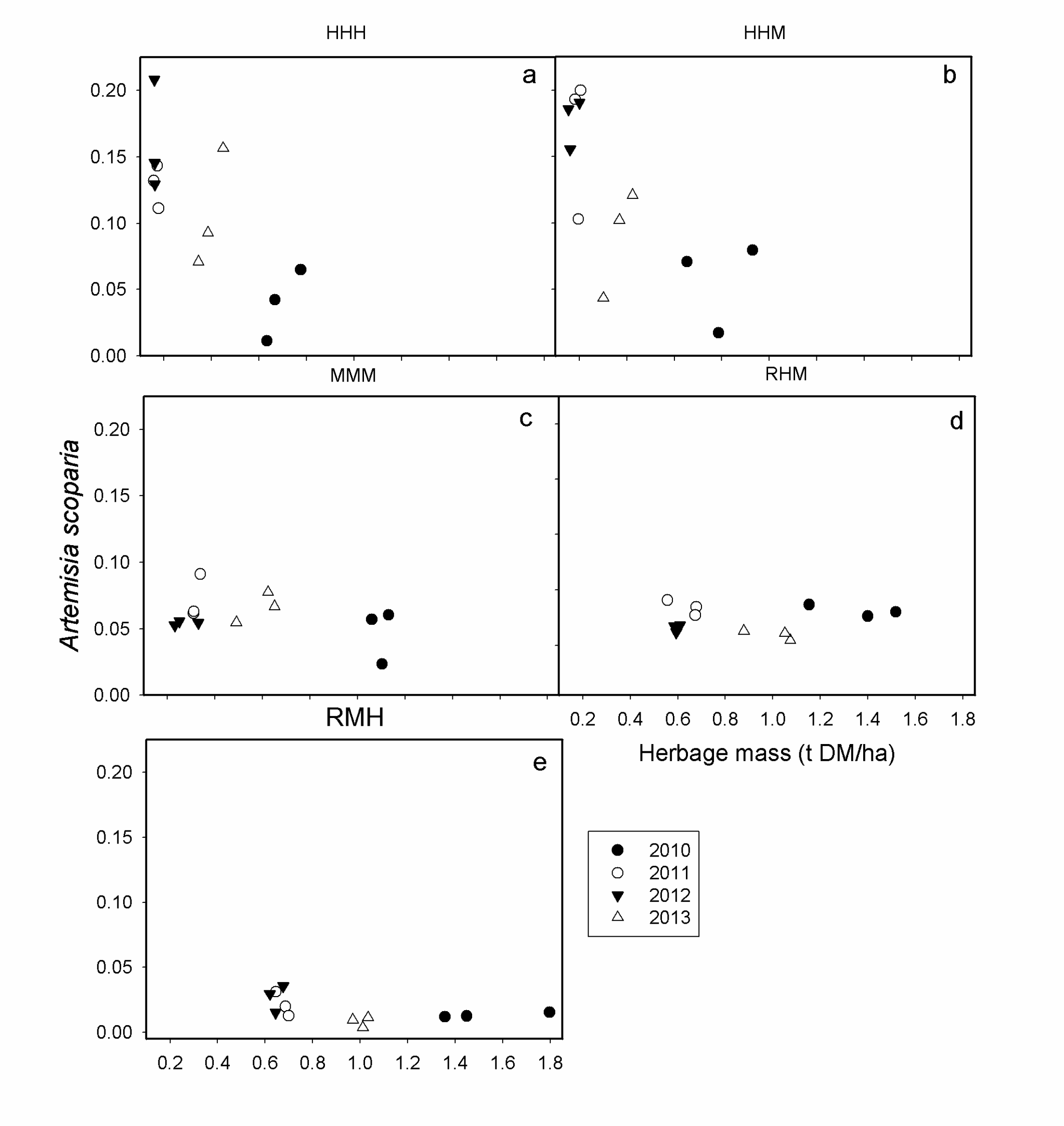

Supplement: Supplementary Information [file srep16434-s1.pdf]
